# Intimate partner violence among women with infertility

**DOI:** 10.1016/S2214-109X(22)00205-4

**Published:** 2022-06

**Authors:** Christine Bourey, Sarah Murray

**Affiliations:** Department of Mental Health, Johns Hopkins Bloomberg School of Public Health, Baltimore, MD 21205, USA

Intimate partner violence (IPV) represents a critical public health problem that impacts mental, physical, and reproductive health throughout the life-course. Infertility is not only an issue of reproductive health, but also a social issue that can influence marital, family, and other interpersonal relationships, particularly in settings where childbearing is highly valued and central to ideas of womanhood. Women experiencing infertility might be socially sanctioned for childlessness or small family sizes in cultures where progeny is among what matters most.^1^ Thus, experiences of violence can affect fertility, and infertility can confer risk for IPV.

Recognising this potential, Yuanyuan Wang and colleagues present a comprehensive systematic literature review and meta-analysis in *The Lancet Global Health* of the prevalence of IPV against women with infertility in nine low-income and middle-income countries (LMICs) in 2000–19.^2^ 30 studies were included in the systematic review, and 25 studies were included in the meta-analysis (7164 participants). This work builds upon previous literature that explores infertility as an under-recognised risk factor for IPV,^3^ undertaking a synthesis that distinguishes IPV from other forms of violence and looks broadly across global regions. The authors used subgroup analyses and meta-regressions to explore variations in IPV prevalence by study period, study region, type of infertility, risk of bias, sample size, and measurement tools. Despite substantial heterogeneity in existing studies, they identified a pooled past-year IPV prevalence of 36·0% (95% CI 20·4–55·2) and lifetime prevalence of 47·2% (31·7–63·3) among women with infertility. It is difficult to compare these prevalence estimates to the general population due to differences in tools used to assess IPV and its subtypes (eg, psychological, physical, and sexual); however, in a small analysis of studies that separately reported IPV prevalence by fertility status, infertile women were more likely to experience physical violence and sexual violence than were fertile women. Lifetime prevalence of psychological and overall IPV did not differ by fertility status.

In the meta-analysis, the authors found that studies conducted after 2010 and in west Asia produced higher lifetime prevalence estimates, and studies with small sample sizes and high risk of bias produced higher past-year prevalence estimates. Notably, there was significant variation among studies that used three common measures (modified Abuse Assessment Screen, WHO Violence Against Women instrument, revised Conflict Tactics Scales), high risk of bias in 16 (of 30) studies, and evidence of publication bias. These findings reiterate a challenge in the broader IPV literature—the impact of measurement on the overall accuracy, comparability, and interpretability of findings—and point to a continued need for local validation studies and assessment of cross-cultural measurement invariance.

Given most studies were hospital-based or clinic-based, we echo the authors’ call for population-based studies. We also agree that studies that expand evidence to additional countries would be beneficial. Despite these limitations on available data, additional data are arguably not required to justify focusing on IPV among women with infertility. From a public health perspective, IPV can perpetuate infertility and compound its psychological consequences.^4,5^ A better understanding is needed of the stigma associated with infertility and how the marginalisation of women with infertility increases the risk of violence and other adverse health and social outcomes. In-depth qualitative research on this topic could help to guide interventions to better support women who experience infertility in settings where varying value and meaning is placed on childbearing and motherhood.

The authors call for interventions at multiple socioecological levels, including individual screening and counselling, dyadic marital counselling, and structural interventions that aim to change economic, politico-legal, physical, and social systems that produce or reproduce IPV risk.^6^ Structural interventions will be crucial to reducing IPV, given the population prevalence of infertility in LMICs (estimated to be as high as 30%) and the need to reduce stigma associated with interventions targeted to women with infertility. Research on structural IPV interventions that not only transform hegemonic masculinities and inequitable gender norms, but also give specific attention to reproductive expectations embedded in the social construction of gender especially, is needed.

As the authors suggest, a gendered lens invariably is important. It is also important, however, that it does not negate focus on the effects of infertility on men. Studies suggest that men experience a loss of social status, social humiliation, or isolation due to infertility,^7^ and multiple theoretical frameworks suggest that this could motivate attempts to reassert power and control in intimate relationships through violence.^8^ Additionally, the power of gendered theories might be augmented when coupled with stigma theories to fully highlight the complex pathways by which experienced marginalisation affects infertility, IPV risk, holistic wellbeing, and their interaction.

Overall, the systematic review by Wang and colleagues brings an important public and human rights issue to the foreground, supplying critical evidence on the prevalence of IPV among women experiencing infertility and bringing the need for additional research and perspectives to the fore.
